# Domestic violence: prevention past due

**DOI:** 10.1093/haschl/qxae034

**Published:** 2024-03-19

**Authors:** Debbie I Chang

**Affiliations:** President and CEO, Blue Shield of California Foundation, San Francisco, CA 94104, United States

**Keywords:** domestic violence, intimate partner violence, prevention, Medicaid

## Abstract

In May 2023, the White House released the National Plan to End Gender-Based Violence, which includes intimate partner or domestic violence (DV). Based on 20 years of experience in California, this commentary provides detailed examples of 2 DV prevention strategies: interrupting intergenerational transmission and addressing macrolevel drivers. Family-strengthening approaches to prevention and justice and increasing economic security are key. Insight into regional policies and programs can inform implementation of the national plan and DV prevention in other states and localities.

## Introduction

In May 2023, the White House released the National Plan to End Gender-Based Violence.^1^ For the first time, a federal administration identified strategies spanning multiple federal agencies for preventing and responding to gender-based violence, including intimate partner or domestic violence (DV). Federal engagement in preventing DV is vital, particularly for supporting the DV prevention at state and local levels that is also essential.

In California, DV prevention began in the 1970s, with early state legislation funding safe housing for survivors. In 1980, the California Alliance Against Domestic Violence (now California Partnership to End Domestic Violence [CPEDV]) was established. The same year, San Francisco was 1 of 9 US cities obtaining a federal grant to address DV. This effort eventually grew into Futures Without Violence (FWV), whose mission is to end violence against women and children.^2^ FWV strives to change social norms about multiple forms of violence through public awareness and education, professional training, policy advocacy, and building sustainable community leadership.^2^ For more than 20 years, the Blue Shield of California Foundation has partnered with CPEDV, FWV, and many other local, state, and national organizations, focusing on preventing and reducing harm from DV in California with an eye towards learning what works at a regional level that could inform national policy.

This commentary highlights evidence-based opportunities based on California's experiences that can be pursued at national, state, and local levels. Two broad strategies address foundational facts about DV. First, family violence typically occurs in a repeating progression from childhood exposure to adult behavior. Of all US children, 27.7% are exposed to physical violence between parents by age 18 years,^3^ and exposed children are up to 4.4 times more likely to become harm-doers as adults.^4^ Most exposure occurs in early childhood because young mothers are at highest risk of experiencing DV.^5,6^ Thus, the first prevention strategy is to interrupt intergenerational transmission of violence.

**Figure 1.**
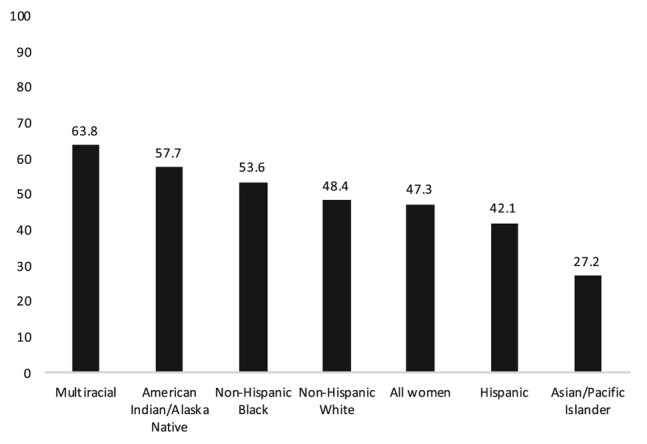
Percentage of surveyed women reporting physical violence, sexual violence, or stalking by an intimate partner during their lifetime, 2016/2017. Source: Leemis RW, Friar N, Khatiwada S, et al. The National Intimate Partner and Sexual Violence Survey: 2016/2017 Report on Intimate Partner Violence. Atlanta, GA: National Center for Injury Prevention and Control, Centers for Disease Control and Prevention; 2022.

Second, DV is a pervasive health equity issue—but not inevitable. In 2016–2017, before DV increased 8% during the COVID-19 pandemic, nearly every other woman in the United States had experienced physical violence, sexual violence, or stalking from an intimate partner during her lifetime.^7^ No one is immune, but Black and Indigenous women disproportionately experience DV (Figure 1). In 2010, the most recent year for which national data are available, women with annual incomes less than $25 000 were 3.5 times more likely to experience DV than those with annual incomes greater than $75 000.^8^ Macrolevel drivers of disparities include deeply rooted social issues, such as unemployment, poverty, gender-based wage inequality, and housing insecurity. The second strategy to prevent DV is to address these upstream factors.

## Interrupting intergenerational transmission

Using a life-course framework is essential to keep DV cycles from repeating within families. Critical periods from birth to age 19 have an outsized impact on adult behavior, highlighting the need to intervene at key times to prevent first instances of violence and interrupt generational cycles.^9,10^

Two-generation approaches including both parents and children are particularly beneficial. Evidence-based home visiting programs for expectant and new parents strengthen families and improve multiple outcomes, including reducing DV.^11^ However, in 2022, the primary federal funding source for home visiting reached 2% of families who could benefit from it.^12^ At least 20 states support home visiting with Medicaid funds^11,13^ and other funding sources. Leveraging Medicaid funding for 2-generation home visiting is a significant opportunity to prevent DV.^13^

Adolescence is another prime period for interrupting intergenerational DV. Peer interactions, antisocial norms, community violence, and experiences of bullying and victimization increase the risk of teen dating violence (TDV). Prevention targeting both adolescents and parents is an effective approach that is especially salient for teens exposed to DV at home.^14^ Of 37 states requiring schools to provide TDV education, 5 states and the District of Columbia include parents in educational programs.^15^ State, local, and school district policies requiring 2-generation, school-based TDV prevention are an underused opportunity to break generational cycles of DV.

Stopping the intergenerational cycle also requires healing from trauma for survivors and exposed children. Healing for 1 generation is prevention for the next. Because populations at highest risk for DV make up a substantial portion of the Medicaid-eligible population, state Medicaid programs are uniquely positioned to support DV prevention across the life course through screening, referral, and ensuring the receipt of needed community and social support services.^16^ The Centers for Medicare and Medicaid Services Innovation Center is making progress in establishing 5 social determinants of health, including interpersonal safety, as screening and quality-of-care indicators.^17,18^

In 2020, California began offering Medi-Cal (Medicaid) providers training, clinical protocols, and reimbursement for screening children and adults for adverse childhood events (ACEs), which include exposure to violence between parents. As of March 2023, ACEs Aware had trained more than 35 000 providers, who had screened more than 1.5 million Medi-Cal members.^19^ Healthy attachment to a primary caregiver mitigates the negative effects of ACEs, but caregivers experiencing DV are less able to be sensitive and responsive to their children.^20^ Dyadic services for children and caregivers promote child social-emotional health and improve maternal parenting and mental health.^20^ Since 2023, Medi-Cal has provided a dyadic behavioral health benefit: behavioral health care for a caregiver that benefits the child can be billed to the child's Medi-Cal identifier.^21^ Other states can leverage these approaches.

Due to the association between childhood exposure to DV and harming intimate partners as an adult, harm-doers also need healing. Prevailing responses to harm-doers focusing on law enforcement and prosecution do not prevent initial or repeating DV.^22^ Many BIPOC (Black, Indigenous, and other people of color) survivors particularly fear involving systems that have often served to oppress them.^23^ A system response addressing healing and prevention is needed.

Restorative justice is a promising multigenerational approach to creating safety for survivors and children by addressing the risk of continued harm, creating supports, and healing the whole family, including the harm-doer.^24^ The 2022 reauthorization of the Violence Against Women Act created the first federal grant program supporting the development of restorative justice options for survivors.^25^ In 2021, California legislation funded pilots of alternative system responses. A promising community-based model is the Collective Healing and Transformation (CHAT) project, designed to stop harm, emphasize survivor safety, and focus on healing in predominantly BIPOC communities in Contra Costa County.^26^ CHAT emphasizes survivor safety and non–law-enforcement options, engages the person who caused harm when appropriate, and includes children as part of the solution. In the first 2 years, 40 cases with 92 individuals showed promise.^27^ CHAT successfully reached those who had not engaged with other DV crisis response and support systems (eg, shelters or police); 81% of participants had never sought help before. More than two-thirds of participants reported the process helped them address conflict and increase safety. CHAT also increased protective factors, reducing the likelihood of future DV.

## Addressing upstream factors

Mitigating biases fostering macrolevel drivers of DV requires rebalancing power and resources. It is essential to align systems involved in state, county, and community DV prevention efforts with the experiences, assets, needs, and priorities of populations most at risk through strengths-based approaches emphasizing protective factors.^1,28^ Examples are available of strengths-based approaches to prevent DV in California Tribal and rural, Latina, and East African communities.^29^

Economic stress and instability increase DV risk and make it harder for women to leave unsafe situations.^30^ In addition, 75%–99% of survivors experience economic abuse^31^ as a harm-doer controls their acquisition, use, or maintenance of economic resources, jeopardizing their economic security and capacity for self-sufficiency.^32^ Improving economic security and mobility for low-income workers and women at risk for and experiencing DV is a pivotal prevention approach.

State Earned Income Tax Credits (EITCs), currently available in 28 states and the District of Columbia, are associated with lower DV rates.^33^ Making state EITCs available across the United States is a key step to improving economic security for populations at risk, along with increasing the uptake of federal and state tax credits. In 2020, 76% of all eligible filers claimed the federal EITC^34^; in 2021, just over half of eligible households claimed the California EITC.^35^ Multichannel, multicultural outreach programs raise awareness among California residents of their eligibility for credits, and multiple volunteer tax information assistance sites help low-income residents with tax preparation.^36^

Paid leave is another cornerstone of economic security. Paid family leave is available to only 6% of workers with earnings in the lowest decile, compared to 43% of the highest decile earners.^37^ Workers without access to paid family leave are disproportionately women and people of color.^38,39^ In 2002, California was the first state to enact paid leave legislation. As of late 2023, paid leave is available to all employees in 8 states and the District of Columbia; 5 states have enacted legislation but benefits are not yet available.^40^ Counties and municipalities have also enacted paid leave laws.^40^ In the current federal policy environment, opportunities for enacting paid leave policies fall to states and smaller jurisdictions.

Economic security and housing stability are inseparable. Housing First is a proven model giving individuals and families access to permanent, stable housing and voluntary services as needed to maximize housing stability.^41^ The Domestic Violence Housing First (DVHF) model also provides flexible economic support directly to survivors.^42^ Many need temporary help with more than just paying the rent, and survivors determine how best to ensure their safety and economic stability. In an early California evaluation of DVHF, 40% of survivors obtained new housing; others used funds to meet urgent needs like car repair.^42^ The DVHF model is more effective than usual services in helping survivors achieve stable housing, safety, and better mental health after 2 years.^43^ State and local DVHF implementation is a key opportunity to change social conditions perpetuating DV.

## Multisector action is key because survivors use multiple systems

The National Plan to End Gender-Based Violence recognizes that prevention requires action across multiple sectors and calls for integrating related goals into programs, policies, and areas not traditionally associated with violence prevention.^1^ Similarly, multisector action is also required at the level of states and smaller jurisdictions.^44^ One goal of multisector action must be to identify and reduce stigma that survivors experience across sectors, limiting their ability to create change for themselves and their children. The strategies described here, which are not exhaustive, include the health care, justice, economic, housing, and employment sectors. Committed philanthropic organizations also play an essential role by helping to spread and scale proven approaches, catalyzing new ones, and evaluating their effectiveness.

Due to the high prevalence of DV, government systems, organizations, and their employees across sectors knowingly or unknowingly interact with survivors who are clients, associates, leaders, and contacts. When ongoing work touches on the deeply rooted social issues entangled with DV, it can also serve to perpetuate or prevent DV.

I close by challenging readers to periodically consider how their work could also help create conditions for breaking the intergenerational cycle of DV and shifting the drivers that make it more likely to occur.

## Supplementary Material

qxae034_Supplementary_Data
